# Symptoms of depression, perceived social support, and medical coping modes among middle-aged and elderly patients with type 2 diabetes

**DOI:** 10.3389/fmolb.2023.1167721

**Published:** 2023-03-20

**Authors:** Chuanyan Zhang, Zezhen Wu, Elna Lopez, Romain G. Magboo, Kaijian Hou

**Affiliations:** ^1^ School of Public Health, Shantou University, Shantou, China; ^2^ Department of Endocrine and Metabolic Diseases, The First Affiliated Hospital of Shantou University Medical College, Shantou, China; ^3^ Graduate School of Shantou University Medical College, Shantou, Guangdong, China; ^4^ Faculty of Graduate School, Lyceum of the Philippines University-Batangas, Manila, Philippines

**Keywords:** type 2 diabetes, depression, middle-aged and elderly patients, glucose metabolism, perceived social support, psychological and behavioral intervention

## Abstract

**Objective:** To understand the prevalence of depression in diabetes population, explore the relationship between diabetes and depression, and the impact of comprehensive psychological and behavioral intervention on depression related to diabetes and glucose metabolism.

**Methods:** 71 middle-aged and elderly patients with type 2 diabetes were investigated and evaluated with Self Rating Depression Scale (SDS), Medical Coping Scale (MCWQ) and Social Support Scale (PSSS). Patients who met the research criteria were randomly divided into an experimental group and a control group. The number of effective cases in the two groups was 36 and 35 respectively. In addition to conventional diabetes drug treatment, the experimental group was supplemented with comprehensive psychological and behavioral intervention, while the control group was only given conventional treatment. The fasting blood glucose, 2-h postprandial blood glucose, body weight and depression index were measured before and after treatment in the two groups.

**Results:** The prevalence of depression in patients with diabetes was as high as 60%, and that in the elderly control group was 5%; In type 2 diabetes population, depression is negatively related to the total score of social support and medical coping surface, and positively related to avoidance, blood sugar, women, course of disease, education level below junior high school, body mass index, and number of complications in medical coping; The fasting blood glucose, 2-h postprandial blood glucose, body mass index, and depression index of the two groups decreased, and the range and speed of decline in the experimental group were higher than those in the control group; There were significant differences between the two groups in fasting blood glucose, 2-h postprandial blood glucose and depression index; During the follow-up period, the blood glucose and depression index of the experimental group increased.

**Conclusion:** Depression has a high prevalence rate in middle-aged and elderly people with type 2 diabetes, and has a negative impact on blood sugar control in diabetes patients; Psychological and behavioral comprehensive intervention can improve the glucose metabolism and depressive symptoms of middle-aged and elderly patients with type 2 diabetes.

## 1 Introduction

Diabetes mellitus is a psychosomatic disease that in the long-term seriously threatens the psychological health of patients, leading to depression, anxiety, cognitive impairment, and other psychological problems, especially in middle-aged and elderly patients with type 2 diabetes, and has attracted extensive attention from endocrinology and clinical psychology at home and abroad. Therefore, paying attention to the psychological health of diabetic patients, studying the effective, comprehensive treatment of diabetes, and improving the quality of life of diabetic patients have become hot topics in medical and clinical psychology research today.

Diabetes mellitus is characterized by chronic and persistent blood glucose elevation, leading to many serious complications. Diabetes mellitus has acomplex etiology, and pathogenesis, and is not yet curable. Therefore, in addition to physical discomfort, diabetes patients must also change their living habits and bear the financial burden of long-term treatment. These are negative life events for patients, threatening their mental health and causing many psychological problems. Depression is one of the most common psychological problems in patients with Diabetes mellitus. In recent years, studies have shown that the prevalence of depression in middle-aged and elderly patients with type 2 diabetes (T2DM) is high, and the number of patients is increasing (Chima et al., 2017; Huang et al., 2018). In 2018, the international prevalence and treatment of diabetes and depression (INTERPRET-DD), a multi-center study completed in 14 countries, found that the prevalence of depression in diabetes patients was 10.6%. According to the Patient Health Questionnaire—9 (PHQ-9), 17% of the middle-aged and elderly patients with type 2 diabetes have moderate to severe depressive symptoms. Women, women with low amount of exercise, low education level, high depression score, and women who had a history of depressive episode were more likely to report depression (Lloyd et al., 2018). Another study reported that mild depression is as common as severe depression in middle-aged and elderly patients (Golden et al., 2017). Additionally, depression in patients with type 2 diabetes will affect their blood sugar control and quality of life, creating a vicious circle.

Psychosocial factors impact the onset, development, and progression of diabetes. There for, the two affect and cause each other. Depression in middle-aged and elderly patients affects their quality of life and their glycemic control. Thus, identifying the factors of diabetes secondary to depression and implementing targeted psychological interventions and behavior modification for diabetic patients can help clinical treatment, reduce medication dosage, improve patient compliance, and enhance the quality of life.

Studies have shown that depressive symptoms and glucose metabolism in middle-aged and elderly patients improve through general psychological support and other interventions (Fiocco et al., 2013; Zimmermann-Schlegel et al., 2019). This study implemented psychological interventions for middle-aged and elderly patients in the following areas: first, behavioral therapy to intervene in diabetic diet and lifestyle changes so that patients can adapt to long-term treatment and strict dietary control as soon as possible; second, supportive therapy and coping guidance to improve the coping skills and social support system of middle-aged and elderly patients; third, relaxation therapy to relieve stress caused by tension-induced blood glucose fluctuations; fourth, cognitive therapy to reduce depression.

The research objective of this study had two levels: to understand the prevalence of depressive symptoms in the middle-aged and elderly patients with type 2 diabetes population, the support they receive from essential populations, and the medical coping style they adopt; and ultimately intend to develop a psychological intervention program to help patients with depressive symptoms and improve their mental health.

## 2 Materials and methods

This study used a descriptive research method. Description information collected included: the profile of the respondents, the differences that existed when grouped according to their profile and their existing relationship with depressive symptoms, and the medical coping styles of middle-aged and elderly patients with type 2 diabetes. The main collection instrument that will be used to measure the variables was a standardized questionnaire using in a Chinese version.

### 2.1 Patient recruitment

The participants of this study included 71 middle-aged and elderly patients (age ≥ 40 years) with type 2 diabetes mellitus who met the diagnostic criteria for diabetes mellitus published by the 2021 American Diabetes Association (ADA) and participated in monthly diabetes health education classes held by the Department of Endocrinology, Longhu People’s Hospital, Shantou, China, from May to July 2022. The survey participants were from the Jinxia community in Longhu District, Shantou, China, and volunteered to participate, excluding older adults with profound physical illnesses (Basic information and clinical data of enrolled patients are shown in Supplementary Table S1).

### 2.2 Study evaluation scale

The study collected patient information using four different standardized questionnaires, as described below.

#### 2.2.1 Interviewee profile

Self-management questionnaire on the general profile of diabetic patients, including name, age, gender, education, fasting glucose, 2-h postprandial glucose, BMI, disease duration, and the number of complications, and incorporating the Depression Self-Rating Scale general profile.

#### 2.2.2 The depression self-rating scale (SDS)

The SDS is a self-assessment scale developed by Zung in 1965 to measure the severity of depression and its treatment changes. The SDS consists of 20 statements and question items, each corresponding to a symptom of interest and rated on a scale of 1–4. The 20 items reflect four specific groups of depressive symptoms: 1) psycho-affective symptoms; 2) somatic disorders; 3) psychomotor disorders; and 4) psychological disorders of depression. The depression severity index rated by the Depression Self-Rating Scale was calculated using the following formula: Depression severity index = cumulative score of each item/80 (maximum total score). The index ranges from 0.25 to 1.0, with indices below 0.5 being no depression; 0.5–0.59 being mild to mild depression, 0.60–0.69 being moderate to severe depression; and over 0.70 being severe depression. Zung tested the reliability and validity of the SDS: its internal consistency was satisfactory, with split correlations of 0.73 (1973) and 0.92 for the odd-even items (1986), with high and moderate correlations with scores on the Beck Depression Questionnaire, the Hamilton Depression Inventory, and the Matrix Metalloproteinase “d” subscale (Zimmermann-Schlegel et al., 2019).

#### 2.2.3 Perceived social support scale (PSSS)

The present study used the Perceived Social Support Scale (PSSS) developed by Xiao Shui in 1986, with minor revisions in 1990. The scale has ten items and consists of three dimensions: objective support (3 items, SS0), subjective support (4 items, SSS), and personal utilization (3 items). Objective support refers to the extent to which an individual has actual contact with others, and subjective support refers to the extent to which an individual feels supported by others. Personal utilization refers to the extent to which an individual can use the support and help of others when experiencing life events. The questionnaire design is basically sound; the items are easy to understand, unambiguous, and have good reliability and validity (Chen et al., 2020).

#### 2.2.4 Medical coping modality questionnaire (MCMQ)

The Medical Coping Questionnaire (MCMQ), developed by Feifell et al. and revised by Jiang Qianjin et al. was used in this study. It is one of the few concise patient-specific coping scales and includes three types of coping strategies-confrontation (or struggle), avoidance, and submission (or acceptance). Factor analysis yielded three factors, confrontation, avoidance, and submission. The correlation coefficients for the three factors were 0.69, 0.60, and 0.76, respectively, with low correlation coefficients between the three factors. After 4 weeks, the retest correlation coefficients for the three-factor scores were 0.66, 0.85, and 0.69 for 36 patients, respectively. This suggests that the reliability and validity of the MCMQ remain satisfactory (Chen et al., 2020).

### 2.3 Study procedure

The first stage: the group test was conducted in a centralized way, and the three questionnaires were filled in at the same time. In the diabetes education class, doctors and interns will cooperate, and the psychological staff will act as the main test. The main test will explain the requirements for filling in the questionnaire to diabetics according to the instructions. The questionnaires will be collected on the spot and checked one by one. The test will take about 60 min. In addition, in the community elderly education center of the hospital, the psychological professional staff served as the main test, and completed the self-assessment depression scale test according to the same procedure.

**FIGURE 1:**
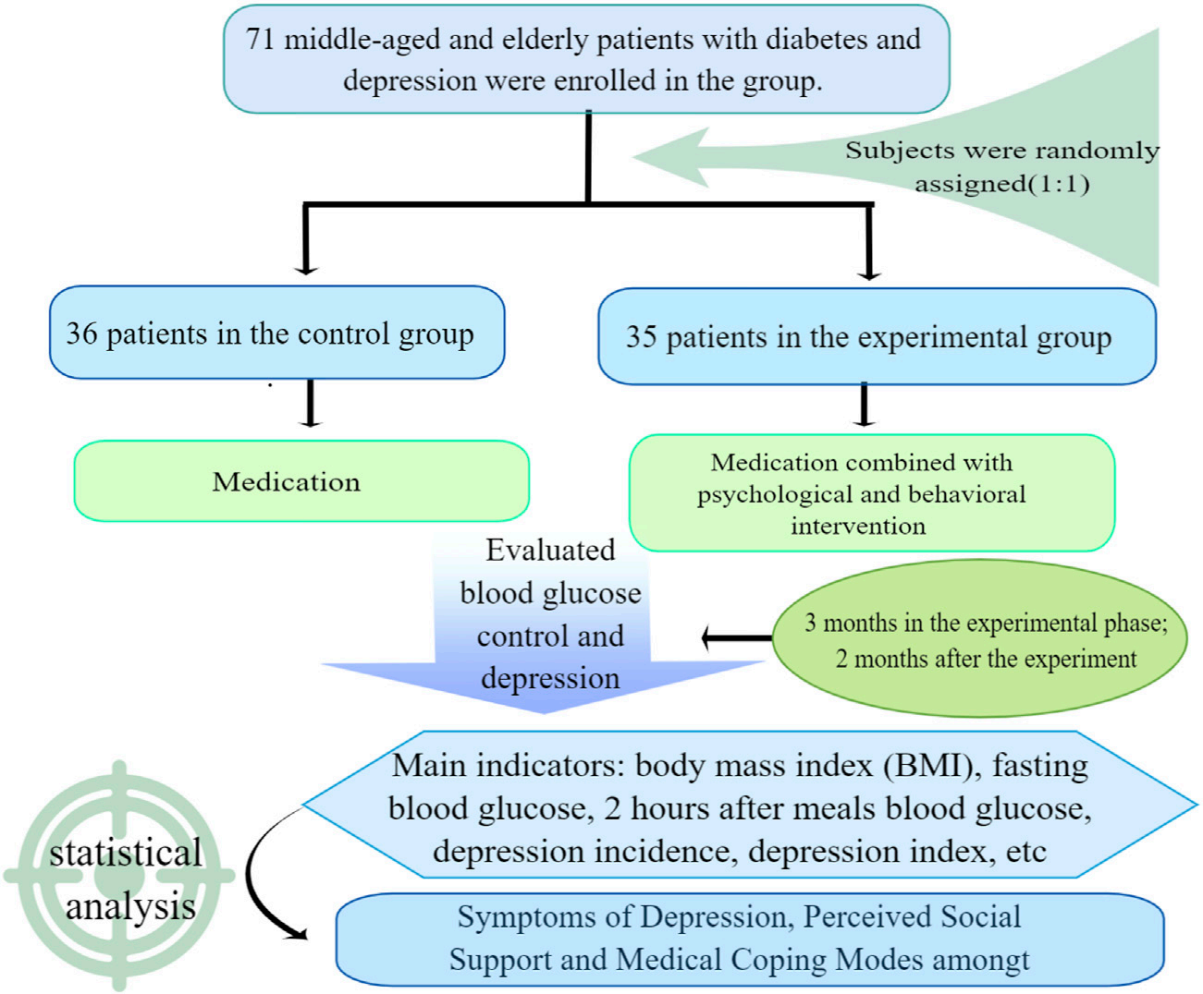
Consort diagram of the study flow.

Through the first stage of research, targeted psychological and behavioral intervention was taken for patients. Seventy one middle-aged and elderly patients with type 2 diabetes who received the questionnaire survey in the early stage were randomly divided into the experimental group (35) and the control group (36). The control group took conventional drug treatment. In addition to conventional drug treatment, the experimental group was supplemented with psychological and behavioral interventions, including supportive therapy, behavioral therapy, cognitive therapy, relaxation therapy, and coping guidance. A 3-month comprehensive psychological and behavioral intervention experiment was conducted, which was called the experimental period. On this basis, a 2-month follow-up observation was followed, which was called the post-experimental follow-up period (Figure 1).

### 2.4 Statistics

Once the respondents’ data were collected from the distributed questionnaires, the data was sent to the statistician for statistical processing. The collected data was entered into Microsoft Excel and processed using SPSS version 2 software. Normally distributed data was calculated based on frequencies and percentages describing the profile of the respondents, weighted averages identifying depressive symptoms, perceived social support, and medical coping patterns of patients with type 2 diabetes. ANOVA was used for multiple group differences, and Pearson’s *r* for correlation between parameters was tested by product difference correlation coefficients.

## 3 Results

### 3.1 The results of the study are presented in tabular forms after statistical and thematic analysis

Table 1 shows that there is no significant difference in gender and education level between the diabetes group and the control group (*p* > 0.05), but there is a significant difference in age between the two groups.

**TABLE 1:** Basic information of the respondent.

	Diabetes group (*n* = 71)	Non-diabetes group (*n* = 97)
Gender (male/female)	31/40	43/54
Age (year)	61.39 ± 10.41	52.52 ± 13.99
Educated above high school	8	13
Educated below high school	63	84

Table 2 shows that the incidence of depressive symptoms and depression in diabetic patients is 60%, and the incidence of depression is significantly higher than in the control group (*p* < 0.001).

**TABLE 2:** Degree of depression in patients with diabetes.

	Depression			Non-depression
	Mild	Moderate	Severe	
Diabetes group (*n* = 71)	25 (35%)***	12 (16%)***	5 (7%)***	29 (40%)***
Non-diabetes group (*n* = 97)	4 (4%)	1 (1%)	0	92 (94.8%)

Note: *p < 0.05, **p < 0.01, ***p < 0.001.

Table 3 shows that the average fasting blood glucose and 2-h blood glucose in the depressed group of diabetes patients are significantly different from those in the non-depressed group (*p* < 0.01). The average fasting blood glucose and 2-h blood glucose in depressed patients are higher than those in non-depressed patients.

**TABLE 3:** Comparison of glucose metabolism between two groups in diabetes patients with or without depressive symptoms.

	FBG (mmol/L)	2HPG (mmol/L)
Depression group (*n* = 42)	9.75 ± 1.03	12.51 ± 1.16
Non-depression group (*n* = 29)	8.69 ± 0.34	11.34 ± 0.74
t	5.32***	4.79***

Note: *p < 0.05, **p < 0.01, ***p < 0.001; FBG: fasting blood glucose; 2HPG: 2-h blood glucose.

Tables 4, 5 show that the factors significantly related to depression in patients with type 2 diabetes include gender, education level below high school, course of disease, number of complications, blood glucose, BMI, total score of social support (SST), utilization of support (SSS), objective support (SSO), and medical coping style of facing and avoiding (*p* < 0.01).

**TABLE 4:** Single factor correlation analysis of depression and possible related factors in patients with type 2 diabetes.

	Gender	Age	Degree of education	Complications	Course of disease
Depression	−0.37**	0.05	−0.13	0.30*	0.48**

Note: *p < 0.05, **p < 0.01, ***p < 0.001.

**TABLE 5:** Single factor correlation analysis of depression and nuclear energy related factors in patients with type 2 diabetes.

	Social support				Medical coping style			Blood glucose		BMI
	SSO	SSS	SSU	SST	Confront	Escape	Surrender	FBG	2hPBG	
Depress-ion	11*	24**	17	23**	46**	397***	241	31**	26**	19*

Note: *p < 0.05, **p < 0.01, ***p < 0.001.

### 3.2 Withdrawal of cases during the experimental period

The number of cases enrolled in March 2022 was 71, with 35 cases in the experimental group and 36 in the control group. After 2 months of treatment, there were 35 cases in the experimental group and 34 cases in the control group; after 3 months, there were 35 cases in the experimental group and 32 cases in the control group. At the fifth month of treatment, there were 33 cases in the experimental group and 32 cases in the control group. Reasons for withdrawal: 2 cases in the experimental group who did not adhere to psychotherapy, 2 cases in the control group whose condition worsened, 1 case of withdrawal from the group due to the medication of the intervention, and 1 case of loss of contact, see Table 6.

**TABLE 6:** Subjects’ general condition.

	Age	Gender (male/female)	Course of disease	Education level (above high school/below high school)
		(Number of cases)	(Year)	(Number of cases)
Experimental group (*n* = 33)	60.36 ± 9.14	15/18	5.48 ± 2.87	4/29
Control group (*n* = 32)	57.86 ± 9.74	15/17	5.63 ± 2.70	4/28

Note: Changes in psychological and biological indicators before and after the psycho-behavioral intervention experiments and during the experimental follow-up period.

As shown in Table 6, there was no significant difference between the experimental group in terms of age, gender, duration of disease and education compared to the control group (all *p* > 0.05). There were no significant changes in psychological and biological indicators before and after the psychological and behavioral intervention experiment and during the follow-up period.

As shown in Table 7, the blood glucose of both the experimental and control groups decreased significantly after treatment. The intra-group differences in fasting blood glucose and 2-h postprandial blood glucose were significant compared with those before treatment (*p* < 0.001), and the differences between the experimental group and the control group in fasting blood glucose and 2-h postprandial blood glucose were significant after 3 months of treatment (*p* < 0.01). In the experimental follow-up after 2 months of stopping the psychological-behavioral intervention period, the blood glucose of the experimental group increased, but the difference was not significant.

**TABLE 7:** Comparison of blood glucose between the two groups before and after treatment and during follow-up (x ± s).

	FBG (mmol/L)	t value			2HPG (mmol/L)	t value	
	Experimental group (*n* = 3)	Control group (*n* = 32)			Experimental group (*n* = 33)	Control group (*n* = 3)	
Before treatment	9.18 ± 0.80	9.23 ± 0.82		0.05	12.13 ± 1.19	11.80 ± 1.00	1.40
The second month	7.57 ± 0.78	8.06 ± 0.85		5.80	10.23 ± 1.29	10.84 ± 1.09	4.34*
The third month	6.66 ± 0.77△	8.24 ± 0.86		60.83***	9.11 ± 1.36△	11.14 ± 1.05	45.41***
Tracking period	8.12 ± 0.23	8.19 ± 0.88		0.15	10.19 ± 1.23	10.80 ± 1.02	4.84*
F value	58.95***	12.61***		-	32.40***	6.33***	-

Note: *p < 0.05, **p < 0.01, ***p < 0.001. △: compared with the index before treatment (*p* < 0.01). FBG: fasting blood glucose; 2HPG: 2-h blood glucose.

As it was shown in Table 8, the depression index of the experimental group continued to decline after treatment, and after 3 months of treatment, the difference between the depression index compared with the pre-treatment was significant (*p* < 0.01), and the difference compared with the control group was also significant (*p* < 0.01). However, a downward trend after 2 months of stopping the psycho-behavioral intervention, but the difference was not significant. The difference between multiple groups before and after treatment of depression index in the control group was insignificant. There was a decreasing trend of BMI in both experimental and control groups before and after the experiment, but the difference was not significant.

**TABLE 8:** Comparison of depression index and BMI between the two groups before treatment, after treatment and during follow-up (x ± s).

	Depression index	t value		BMI
	Experimental group (*n* = 33)	Control group (*n* = 32)		Experimental group (*n* = 33)
Before treatment	0.56 ± 0.11	0.55 ± 0.09	0.25	25.85 ± 1.47
The second month	0.51 ± 0.10	0.53 ± 0.10	0.45	25.75 ± 1.55
The third month	0.41 ± 0.09△	0.52 ± 0.09	28.57***	25.23 ± 1.51
Tracking period	0.50 ± 0.091	0.51 ± 0.09	0.28	25.55 ± 1.47
F value	14.71***	1.10	-	1.03

Note: *p < 0.05, **p < 0.01, ***p < 0.001. △: compared with the index before treatment *p* (0.01).

## 4 Discussion

This study is in line with the results of other domestic studies (Dunstan et al., 2017; Chen et al., 2020), further confirming the high incidence of depression in diabetic patients. Type 2 diabetic patients are primarily elderly, and the elderly have a high prevalence of depression due to their social environment and physiological condition, so this study set up an elderly control group without serious diseases. The results showed that the difference between the depression rate in the diabetic group and the control group was significant [*p* (0.001)]. The two possible reasons for depression due to diabetes are:

There is a biological correlation between diabetes mellitus and depression. A deficiency causes depression in the function of 5-hydroxytryptamine and norepinephrine. In contrast, in an animal model of diabetes, it was found that intravenous administration of 5-hydroxytryptophan in mice lowered blood glucose but had no effect on insulin secretion. 5-hydroxytryptophan, a glucose-regulatory effect unrelated to insulin, can be blocked by 5-hydroxytryptophan antagonists and 5-hydroxytryptophan also decreases feeding behavior in the proportion of carbohydrates and total energy intake (Hasan et al., 2014). It is suggested that both diabetes and depression are correlated with 5-hydroxytryptamine, but their specific pathophysiological relevance needs further study. Diabetes, as a chronic disease, means lifelong treatment, changes in lifestyle habits, and long-term medical expenses, which are traumatic life events for patients, thus causing depression.

As known from Table 3, the differences in fasting blood glucose and 2-h postprandial blood glucose between depressed and non-depressed groups are significant, indicating that depression affects diabetic patients’ glucose metabolism, which is consistent with the findings of Heather et al. (Roy and Lloyd, 2012), but the mechanism is not precise, and there may be three reasons: (1) there is a biological mechanism related to depression and diabetes, and the abnormal secretion of hormones related to depression may affect metabolic control of diabetes; (2) depression may affect the compliance of diabetic patients, such as patients do not follow medical advice, do not control their diet, do not exercise, etc. (Carlbom et al. (2017)), and indirectly affect the metabolic control of diabetes; (3) depression belongs to bad mood, which is a chronic psychological stress, while depression itself affects the cognitive ability of patients, cognitive ability usually includes attention, memory, analytical and synthesis ability, etc. (de Vries McClintock et al. (2016)), which is the main view at present. The cognitive ability usually includes attention, memory, analytical and synthesis ability, etc. These are the basis for perceiving the surrounding environment, evaluating life events, and dealing with problems. Magnuson et al. (2019) further confirmed that depression is the primary correlate of cognitive dysfunction in Middle-aged and elderly patients with type 2 diabetes. Cognitive dysfunction can cause cognitive biases in patients, increasing the impact of possible adverse life events and further aggravating psychological stress. In stressful situations, cortisol is hypersecretion, and large amounts of cortisol can reduce muscle glucose utilization and promote gluconeogenesis to increase blood glucose. Secondly, cortisol can antagonize insulin and inhibit blood glucose catabolism, causing an increase in blood glucose (Tyagi et al., 2021). As shown in Tables 1, 2, although depression and diabetes exist in different specialties, they are both biologically intrinsically linked and interact with each other during the long course of the disease. Diabetes causes depression, decreasing the patient’s quality of life and making it difficult to control the patient’s blood glucose, forming a vicious circle.

Social support as an essential intermediate variable affecting the outcome of stress response generally reduces the stress response, and many quantitative studies have demonstrated that social support is negatively associated with the stress-induced psychosomatic response (Liu et al., 2021). Secondly, low social support alone can lead to adverse psychological experiences in individuals, such as feelings of loneliness and helplessness, and social support can maintain good emotional experiences in patients (Bedaso et al., 2021). This study showed that total social support scores were negatively correlated with depression scores in middle-aged and elderly patients with type 2 diabetes, which suggests that social support can improve depression and maintain a good mood in diabetic patients. This result is consistent with the view mentioned above. This study further showed that the utilization of support and subjective support was negatively and significantly correlated with depression. This suggests that individuals’ degree of social support is influenced by various factors, such as individual cognitive factors affecting the quality of access to subjective support in social support; and individual coping styles affecting the utilization of support. In a study of diabetes combined with depression, 637 elderly patients with hypertension and diabetes aged 65 years and over in Sichuan Province, China were collected. Structural equation model (SEM) was used to test the hypothetical relationship between variables. It was found that social support was indirectly negatively related to weakness in diabetes and depression, reducing the weakness of elderly patients with hypertension and diabetes while alleviating depression (Liu et al., 2020). Therefore, improving the objective status of social support and enhancing the subjective experience of being supported by individuals with diabetes have direct implications for alleviating depression in diabetes.

Coping is an important intermediate variable affecting the outcome of a stress response. Generally speaking, positive coping can reduce stress and benefit health, while negative coping aggravates stress and is detrimental to health. During the period of COVID-19, Ding Xiushi et al. randomly divided 150 middle school students with anxiety scores greater than 50 who volunteered to participate in the intervention experiment into the control group and the intervention group, and conducted 8-week peer education intervention based on model 32 for the intervention group. After intervention, the score of Self Rating Anxiety Scale (SAs) in the intervention group was better than that in the control group (*p* < 0.001). In addition, the scores of self-rating depression scale (SDS) of the two groups of patients decreased, but the effect of the intervention group was more significant (*p* < 0.001), indicating that active coping and intervention can reduce the level of depression in adolescents (Ding and Yao, 2020). However, the relationship between coping and illness measured using the Medical Coping Style was inconsistent with this pattern. The Medical Coping Style Questionnaire is a coping scale used by patients to measure their coping processes in response to a life event such as an illness. It has been suggested that different medical coping styles can decrease or increase the stress response in different diseases. In contrast, the same medical coping style may reduce stress response in one disease and increase it in another disease instead (Sotnikov et al., 2014). Avoidance coping among medical coping styles was highly negatively correlated with psychosomatic symptoms of cancer patients, suggesting that avoidance may reduce the effect of psychological stress and thus improve psychosomatic symptoms of cancer patients. In contrast, confrontation coping styles, which are positive coping, were not significantly correlated with psychosomatic conditions of cancer patients (Taniguchi and Mizuno, 2016). The results of this study showed that among the three coping styles of confrontation, submission, and avoidance, depressive symptoms in diabetic patients were negatively correlated with the medical coping style of confrontation. It indicates that the adoption of the confrontation style of coping by diabetic patients in the course of the disease would contribute to the alleviation of depressive symptoms; submission was positively correlated with depression in diabetic patients, indicating that the adoption of the medical coping style of submission to the fact of having diabetes was the main cause of depression. While avoidance is a negative coping style in general, this study showed that avoidance was not associated with depressive symptoms in diabetic patients.

Depressive symptoms in middle-aged and elderly patients with type 2 diabetes were positively correlated with blood glucose, disease duration, the number of complications, body mass index, and female, i.e., the higher the fasting blood glucose, 2-h postprandial blood glucose, the longer the disease duration, the more complications, and the more obese, the more severe the depression. In contrast, middle-aged and elderly female diabetic patients were more likely to have depressive symptoms, consistent with existing studies (Silverman et al., 2015; Adams et al., 2016). In this study, depression was positively correlated with an education level below high school. Presumably, because of the low education level, the receptiveness of diabetes knowledge was affected: easy to listen to rumors, did not form a correct understanding of diabetes, lack of treatment confidence, and showed more severe depressive symptoms. However, this result is inconsistent with other studies (Noh et al., 2019; Sharma et al., 2021). They concluded that depression was positively correlated with an education level above high school, subject to further validation studies in the future. In clinical practice, clinicians can improve the recognition of depression in diabetes combined with these characteristics and also focus on women, the long duration of disease, more complications, high fasting glucose and 2-h postprandial glucose, and obese diabetic patients during psycho-behavioral interventions.

In this study, we compared the efficacy between the experimental and control groups through a psycho-behavioral intervention test in the experimental group of middle-aged and elderly patients with type 2 diabetes at the second and third months of the treatment and 2 months after the end of the psychological intervention. We found that the fasting and 2-h postprandial blood glucose of both the experimental and control groups decreased significantly after 2 months of treatment. However, the rate of decrease was faster in the experimental group, which indicated that the glucose-lowering effect of conventional diabetes treatment is still ideal. However, the psycho-behavioral intervention also played an auxiliary role in glucose-lowering, and this result is consistent with other reports in the literature (Zheng et al., 2019; Liu et al., 2022). From the observation of the 3-month treatment, psycho-behavioral interventions were found to have a short-term effect on improving glucose metabolism in patients with type 2 diabetes. Psycho-behavioral therapy is a comprehensive stress management technique that effective and decreases stress levels. As mentioned earlier, the blood glucose of patients under stress will be elevated through neuroendocrine mechanisms. In healthy organisms, this elevation will be normalized more quickly through the organism’s regulation. However, in Middle-aged and elderly patients with type 2 diabetes, due to the pathological mechanism of relative or absolute insulin deficiency, the stress-induced elevation of blood glucose cannot be metabolized appropriately. The psycho-behavioral therapy can reduce the effect of stress on diabetic patients and help them control their blood glucose. At the same time, this study also introduced behavioral therapy into diet and exercise therapy in conventional diabetes treatment and used positive reinforcement to cultivate diabetic patients’ lifestyles that are beneficial to treatment and consolidate the efficacy of diet and exercise therapy.

**FIGURE 2:**
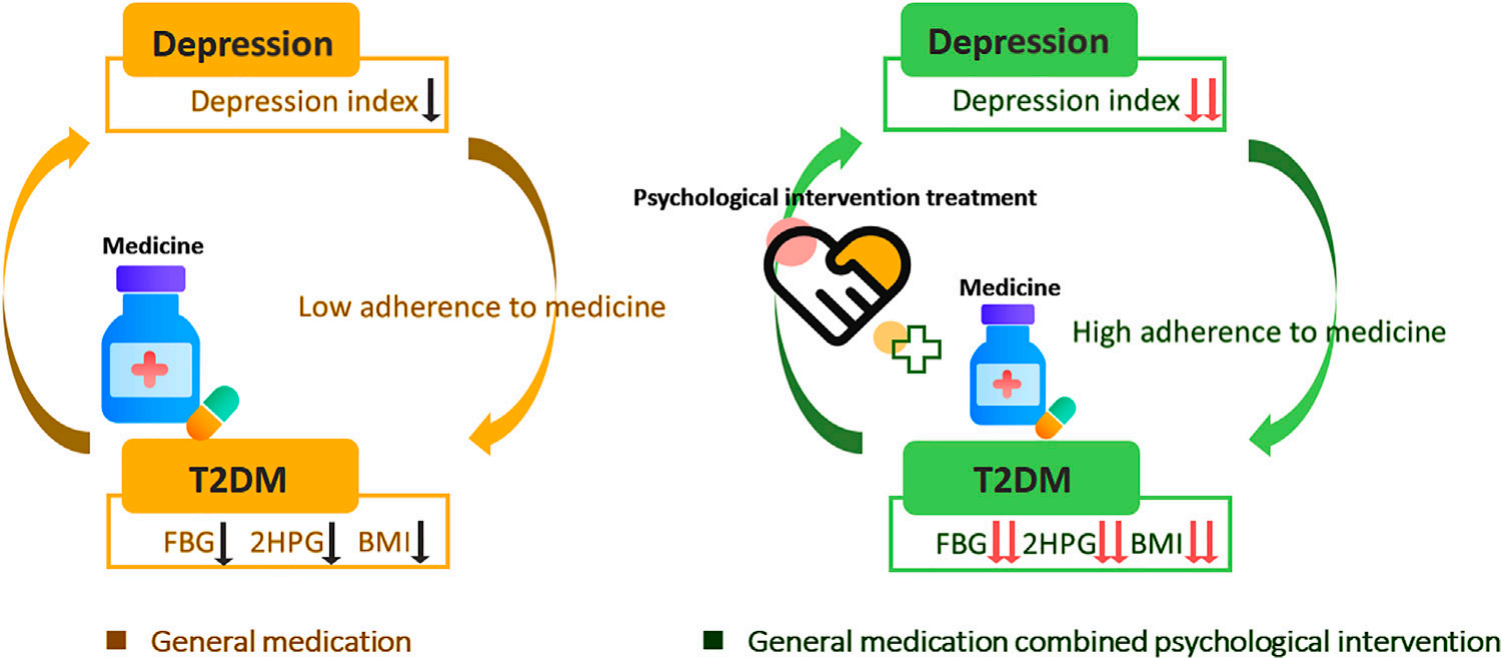
Medication combined with psychological intervention in depression patients with T2DM. FBG, Fasting blood-glucose; 2HPG, 2-h post-meal blood glucose; BMI, Body Mass Index.

After 3 months of treatment, there was a significant decrease in the depression index of diabetic patients in the experimental group, indicating that the psycho-behavioral intervention effectively alleviated the middle-aged and elderly patients with type 2 diabetes’ depressive symptoms. Study shows that incorrect cognition of life events causes depression (Saleh et al., 2017). This study used cognitive therapy to correct patients’ misperceptions about diabetes, mainly by consolidating diabetes knowledge, allowing patients to correctly understand the characteristics of diabetes, establishing treatment confidence, and adopting a confronting response to the fact that they have diabetes. At the same time, general psychological support for patients was strengthened. Through the guidance of a general coping style, the use of support by patients is improved so that patients can maintain good emotions and improve their compliance with treatment, which is conducive to the relief of depression. Although the depression index decreased through psycho-behavioral intervention, the difference between groups was significant only after 3 months, which means that changing patients’ cognition and coping style needs a long-time process. It may be the change of monoamines such as 5-hydroxytryptamine, norepinephrine and their receptors in the brain of long-term depressed patients cannot be restored in a short time, just as the efficacy of antidepressants usually takes 3 months to take effect (Israelyan et al., 2019). The significant effect of psycho-behavioral interventions is not immediately apparent because the pathological changes that have occurred need a process to return to normal. There was also a decrease in the depression index in the control group, suggesting that the simple decrease in blood glucose also contributed to the relief of depressive symptoms in diabetic patients (Figure 2).

It is clear from the first phase of the study that middle-aged and elderly patients’ diabetes and depressive symptoms interact, with diabetes itself causing depressive symptoms to occur or worsen and depression affecting the metabolic control of diabetes, forming a vicious circle. Therefore, improving depressive mood can relieve patients’ emotional disorders, enhance patients’ confidence in facing the disease, and improve patients’ quality of life. It also promotes blood glucose control, indicating that human is a biological-psychological-social organic whole. Biological and psychosocial factors are constantly interconnected and influenced by each other, so it is not enough to control the metabolism of diabetes in diabetes treatment only by drugs, etc., (Almutairi et al., 2020; Que et al., 2021; Wu et al., 2023). Therefore, attention to the depression of patients and providing timely and effective psycho-behavioral interventions are necessary for the comprehensive treatment of diabetes mellitus.

BMI was a factor significantly associated with depression in the first phase of the study. In our study, as the depression index decreased, the BMI also decreased. However, the difference was not significant, probably because the total observation period was only 5 months, which was too short. In the experimental group, 2 months after the end of the psycho-behavioral intervention, the 2-h postprandial blood glucose, and depression index all tended to increase. Compared with the control group, the situation of blood glucose in the experimental group was not better. This finding indicates that although the short-term effect of the psychological and behavioral intervention is ideal, after the interventions stopped, the newly learned behavior, coping style, and lifestyle of patients through intervention will slowly subside in the absence of reinforcement and support, which suggests that psychological and behavioral intervention should be carried out for a long time. Although current diabetes education includes psychological support, it is limited to general empathy, comfort, and encouragement. It remains to be explored how professional monitoring of psychological problems and psycho-behavioral interventions can be part of the routine treatment of diabetes in the clinical setting. This also raises thoughts for the shift from a bio-medical model to a bio-psycho-social medical model, which should attract the attention of clinicians and healthcare.

## 5 Shortcomings of this study

Due to the limitation of objective conditions, the sample size was not large enough, and the experimental period was relatively short. Whether the results obtained are representative and generalizable needs further validation studies. Depression in middle-aged and elderly patients with type 2 diabetes is often combined with anxiety; the two are interrelated and affect each other. Anxiety can also affect the metabolic control of diabetes, whether it will impact the experimental results to be confirmed by further studies in the future.

## 6 Conclusion

The incidence of depression in middle-aged and elderly patients with type 2 diabetes is 60%, which is significantly higher than that in the healthy middle-aged and elderly population, so it is necessary to pay attention to the mental health of diabetic patients and provide corresponding psycho-behavioral interventions.

The occurrence of depression in patients with type 2 diabetes is related to various factors. It is suggested that high blood glucose, long disease duration, the high number of complications, obese people, and women should be the focus of psychological intervention. Secondly, lowering blood glucose, correcting the patient’s surrender coping style, helping the patient to establish a coping style to face the disease, and providing more social support can help alleviate the depression.

The depressive disorder of middle-aged and elderly patients with type 2 diabetes forms a vicious circle with blood glucose control, so clinical work should provide comprehensive and multi-latitude treatment for diabetic patients, to control blood glucose and treat depression.

Comprehensive psycho-behavioral interventions can effectively relieve middle-aged and elderly patients’ depressive symptoms, assist in lowering glucose, and interrupt the vicious cycle of depressive symptoms leading to poor glycemic control and hyperglycemia, leading to depressive symptoms.

The immediate effect of psycho-behavioral interventions is noticeable. However, the long-term effect decreases, suggesting that psycho-behavioral interventions should become part of the routine treatment of diabetes, and hospitals and clinicians should pay attention.

## Data Availability

The original contributions presented in the study are included in the article/Supplementary Materials, further inquiries can be directed to the corresponding author.

## Supplementary material

SUPPLEMENTARY TABLE S1 Basic information and clinical data of 71 enrolled patients.
